# Identification of microRNAs specific for high producer CHO cell lines using steady-state cultivation

**DOI:** 10.1007/s00253-014-5911-4

**Published:** 2014-07-23

**Authors:** Andreas Maccani, Matthias Hackl, Christian Leitner, Willibald Steinfellner, Alexandra B. Graf, Nadine E. Tatto, Michael Karbiener, Marcel Scheideler, Johannes Grillari, Diethard Mattanovich, Renate Kunert, Nicole Borth, Reingard Grabherr, Wolfgang Ernst

**Affiliations:** 1 Austrian Centre of Industrial Biotechnology (ACIB GmbH), Muthgasse 11, 1190 Vienna, Austria; 2 Department of Biotechnology, VIBT—BOKU—University of Natural Resources and Life Sciences Vienna, Muthgasse 18, 1190 Vienna, Austria; 3 Department of Food Science and Technology, VIBT—BOKU—University of Natural Resources and Life Sciences Vienna, Muthgasse 18, 1190 Vienna, Austria; 4 School of Bioengineering, University of Applied Sciences FH-Campus Vienna, Muthgasse 56, 1190 Vienna, Austria; 5 Institute for Genomics and Bioinformatics, Graz University of Technology, Petersgasse 14, 8010 Graz, Austria

**Keywords:** Chinese hamster ovary cells, Productivity, Chemostat, Microarray, miRNA expression profiling, miRNA target identification

## Abstract

MicroRNAs are short non-coding RNAs that play an important role in the regulation of gene expression. Hence, microRNAs are considered as potential targets for engineering of Chinese hamster ovary (CHO) cells to improve recombinant protein production. Here, we analyzed and compared the microRNA expression patterns of high, low, and non-producing recombinant CHO cell lines expressing two structurally different model proteins in order to identify microRNAs that are involved in heterologous protein synthesis and secretion and thus might be promising targets for cell engineering to increase productivity. To generate reproducible and comparable data, the cells were cultivated in a bioreactor under steady-state conditions. Global microRNA expression analysis showed that mature microRNAs were predominantly upregulated in the producing cell lines compared to the non-producer. Several microRNAs were significantly differentially expressed between high and low producers, but none of them commonly for both model proteins. The identification of target messenger RNAs (mRNAs) is essential to understand the biological function of microRNAs. Therefore, we negatively correlated microRNA and global mRNA expression data and combined them with computationally predicted and experimentally validated targets. However, statistical analysis of the identified microRNA-mRNA interactions indicated a considerable false positive rate. Our results and the comparison to published data suggest that the reaction of CHO cells to the heterologous protein expression is strongly product- and/or clone-specific. In addition, this study highlights the urgent need for reliable CHO-specific microRNA target prediction tools and experimentally validated target databases in order to facilitate functional analysis of high-throughput microRNA expression data in CHO cells.

**Electronic supplementary material:**

The online version of this article (doi:10.1007/s00253-014-5911-4) contains supplementary material, which is available to authorized users.

## Introduction

Chinese hamster ovary (CHO) cells are the most frequently applied expression system for the production of therapeutic proteins, mainly because of their ability to grow in suspension and to secrete complex recombinant proteins that are correctly processed. CHO cells allow proper protein folding and post-translational modifications such as human-like glycosylation which might be required for biological efficacy. Mammalian cells have originally been considered as the least effective production systems. But due to the advances in cell culture technology over the last three decades, product titers of 1 to 5 g L^−1^ are typically reached in industry today (Hacker et al. 2009). This was mainly achieved by vector, media, and process optimizations, but also, cell engineering has been applied to improve the productivity of recombinant CHO cells. A multitude of strategies to engineer apoptosis resistance, cell proliferation, product secretion, or cell metabolism have been described and are comprehensively reviewed elsewhere (Kim et al. 2012). These approaches often include the stable overexpression of one or more genes, which constitutes an additional burden to the translational machinery of the cell. To circumvent this drawback, microRNAs (miRNAs) have been considered as potential targets for cell engineering (Müller et al. 2008). MicroRNAs are short (~22 nt) endogenous RNAs that play an important role in the regulation of gene expression (Bartel 2004). They are predominantly transcribed by RNA polymerase II, processed by Drosha and exported to the cytoplasm where the ~70-nt precursor hairpin (pre-miRNA) is cleaved by Dicer resulting in a miRNA/miRNA* duplex. One strand (mature miRNA) associates with argonaute (AGO) proteins and forms a miRNA-induced silencing complex (miRISC) which recognizes the target messenger RNAs (mRNAs) predominantly by binding to the three prime untranslated region (3′ UTR) through imperfect base pairing (Krol et al. 2010). MicroRNAs act in a post-transcriptional manner by decreasing translational efficiency and/or transcript levels. A single miRNA can repress hundred different mRNAs and thereby regulates entire gene networks (Hobert 2008). They play crucial roles in a wide range of biological processes including development, proliferation, differentiation, apoptosis, and metabolism (Bartel 2004; He and Hannon 2004). In mammalian cells, miRNAs are predicted to regulate or fine-tune gene expression of ~50 % of all protein-coding genes (Krol et al. 2010). In CHO cells, it has already been shown that miRNAs can be utilized to improve growth (Jadhav et al. 2012), apoptosis resistance (Druz et al. 2013), and specific productivity (Barron et al. 2011; Jadhav et al. 2014; Strotbek et al. 2013).

In this study, we analyzed and compared the miRNA expression pattern of high, low, and non-producing recombinant CHO cell lines to identify miRNA targets that are involved in recombinant protein synthesis and secretion and thus might be promising starting points for cell engineering to increase specific productivity. Cross-species miRNA microarrays were used as screening tools and quantitative reverse transcription polymerase chain reaction (qRT-PCR) for the confirmation of differentially expressed miRNAs. Samples for comparative physiological analyses are generally taken in the exponential growth phase. However, the cellular transcriptome of mammalian cells is very dynamic during batch cultivation where the conditions change continuously due to nutrient consumption and the accumulation of metabolites (Hernandez Bort et al. 2012; Koh et al. 2009). For this reason, we applied steady-state cultivation using a continuous process (chemostat). This enabled the cultivation of the cells with a defined specific growth rate in a constant environment.

Because miRNAs regulate gene expression via interaction with their target mRNAs, identifying targets is crucial for understanding the biological function of miRNAs. However, although more than 400 expressed mature miRNAs have been identified in CHO cells (Hackl et al. 2012), the exact biological function of most of them in cultivated cells is still largely unknown. To identify potential miRNA-mRNA interactions, miRNA expression data were linked to mRNA expression data from microarray analysis. But due to a lack of reliable computational prediction tools and CHO-specific experimentally validated miRNA target databases, high-throughput miRNA target identification remains a major challenge.

## Material and methods

### Cell lines

Recombinant CHO suspension cell lines expressing the 3D6 single-chain Fv-Fc fusion antibody (3D6scFv-Fc) and human serum albumin (HSA) with low and high productivities were established as previously described (Maccani et al. 2014). Briefly, protein-free cultivated dihydrofolate reductase deficient CHO cells (DUKX-B11, ATCC CRL-9096) were used as host cell line. After cotransfection with a pCI-neo mammalian expression vector (Promega, Madison, WI, USA) containing the appropriate gene of interest and a second plasmid (p2-dhfr) which contains the dihydrofolate reductase gene, stable recombinant cells were selected in the presence of G418 and the absence of hypoxanthine and thymidine. Productivity was improved by stepwise increase of the methotrexate (MTX) concentration and two steps of subcloning by limiting dilution. Low-producing cell lines (CHO 3D6scFv-Fc low producer and CHO HSA low producer) were selected at 0.1 μM MTX and high-producing cell lines (CHO 3D6cFv-Fc high producer and CHO HSA high producer) at 0.4 μM MTX. A stable non-producing cell line (CHO empty vector) was established by cotransfection of the host cell line with the empty pCI-neo vector and p2-dhfr. MTX concentration was increased to 0.1 μM.

### Steady-state cultivation

Chemostat cultivations were conducted in 800-mL cell culture bioreactors (DS0700TPSS, DASGIP, Jülich, Germany). The inocula were expanded in spinner flasks starting from the working cell bank. Exponentially growing cells from passage six were used for inoculation. The initial cell concentration was 2.5 × 10^5^ cells mL^−1^. The cultures were maintained at 37 °C, pH 7.0, 30 % dissolved oxygen, and an agitation speed of 80 rpm. The medium was composed of DMEM without glucose and Ham’s F12 (1:1) supplemented with 4 mM L-glutamine, 0.25 % soy peptone (Quest International, Naarden, The Netherlands), 0.1 % Pluronic F68, and a protein free supplement (Polymun Scientific, Klosterneuburg, Austria). After 3 days of batch cultivation, the process was switched to continuous operation for 11 days. Fresh medium was supplied at a constant flow rate to maintain a dilution rate D of 0.5 d^−1^. The working volume was kept constant at 400 mL using a DASGIP level sensor. Samples for off-line monitoring were taken once a day. D-glucose, L-glutamine, L-glutamate, and ammonium concentration were measured with a bioprofile analyzer (BioProfile 100 Plus, Nova Biomedical, Waltham, MA, USA). Cell concentration was determined by counting the nuclei of lysed cells with a Z2 Coulter Counter (Beckman Coulter, Brea, CA, USA). Cell viability was determined by trypan blue exclusion using a hemocytometer.

### Productivity determination

The concentrations of the secreted products were determined from the culture supernatants using sandwich ELISA assays as previously described (Maccani et al. 2014). Briefly, 96-well microtiter plates (Nunc MaxiSorp, Thermo Fisher Scientific, Waltham, MA, USA) were coated with 0.33 μg mL^−1^ goat anti-human IgG (γ-chain specific) antibody (I3382, Sigma-Aldrich, St. Louis, MO, USA) to detect 3D6scFv-Fc. Affinity purified 3D6scFv-Fc was used as a standard. Standard and samples were applied in serially twofold dilutions, and captured 3D6scFv-Fc was incubated with 0.5 μg mL^−1^ horseradish peroxidase conjugated goat anti-human IgG (γ-chain specific) antibody (62–8420, Life Technologies, Carlsbad, CA, USA). Staining was conducted using *o*-phenylenediamine and H_2_O_2_. The absorption was measured at 492 nm with an infinite M1000 microplate reader (Tecan, Männedorf, Switzerland). HSA concentrations were determined using the Human Albumin ELISA Quantification Set (E80-129, Bethyl, Montgomery, TX, USA) according to the manufacturer’s instructions.

The specific product secretion rate q_P_ (pg cell^−1^ d^−1^) during steady-state cultivation was calculated according to Eq (1), where D (d^−1^) represents the dilution rate. VCC (cells mL^−1^) is the viable cell concentration and C_P_ (μg mL^−1^) the product concentration.

1 $$ {\mathrm{q}}_{\mathrm{p}}=\mathrm{D}\times \frac{{\mathrm{C}}_{\mathrm{p}}}{\mathrm{VCC}}\times {10}^6 $$

### RNA isolation

Total RNA samples were isolated from 5 × 10^6^ cells using the Ambion TRI Reagent (Life Technologies, Carlsbad, CA, USA) according to the manufacturer’s instructions using chloroform for extraction. Yield and purity were determined using the NanoDrop 1000 sprectrophotometer (Thermo Fisher Scientific, Waltham, MA, USA). Only total RNA samples with an A260/A280 ratio between 1.8 and 2.0 and an A260/A230 ratio >2.0 were used in this study. The integrity of the RNA samples was analyzed using the Agilent 2100 Bioanalyser together with the RNA 6000 Nano LabChip kit (Agilent, Santa Clara, CA, USA). The RNA integrity number (RIN) was ≥9.9 for all samples which indicates a very high sample quality.

### MicroRNA microarray

Cross-species microRNA microarray assays were conducted as described previously (Hernandez Bort et al. 2012). Briefly, epoxy-coated Nexterion glass slides were spotted with eight replicates of a locked nucleic acid (LNA) probe set consisting of 2,367 probes against human, mouse, and rat miRNAs based on miRBase 16. Total RNA extracts of three biological replicates per cell line from independent steady-state cultivations were analyzed. Therefore, 800 ng total RNA were hybridized against a common reference (pooled RNA from all samples). To label the miRNAs, the Exiqon Power Labeling Kit (Exiqon, Vedbaek, Denmark) was used according to the manufacturer’s instructions. The arrays were hybridized for 16 h at 56 °C followed by automated washing and drying with nitrogen using a Tecan HS 400 hybridization station (Tecan, Männedorf, Switzerland). Slides were then scanned at 10-μm resolution and auto-gain settings using a Roche NimbleGen MS200 scanner (Roche NimbleGen, Madison, WI, USA).

Feature extraction was conducted using the GenePix software (Molecular Devices, Sunnyvale, CA, USA). The LIMMA package of R/Bioconductor was applied for background correction, normalization, and statistical analysis as previously described (Hackl et al. 2010). The resulting *p * values were corrected for multiple testing according to Benjamini and Hochberg (Benjamini and Hochberg 1995). Raw and normalized microarray data have been deposited in NCBI’s Gene Expression Omnibus (GEO) database (www.ncbi.nlm.nih.gov/geo/) and are available under accession number GSE57023.

The software Genesis 1.7.6 (Sturn et al. 2002) was used to conduct hierarchical clustering.

### mRNA microarray

As microarray platform, the 4 × 44 k design from Agilent (CA, Santa Clara, USA) was chosen. Sixty-mer oligonucleotide probes were designed based on the published genomic sequence of the CHO-K1 cell line (Xu et al. 2011). The probe set and array design (20,650 genes, spotted in duplicates) were submitted to the Agilent eArray platform.

Total RNA extracts of three biological replicates per cell line from independent steady-state cultivations were analyzed in duplicates (dye swap). The Agilent Low Input Quick Amp Labeling Kit was used to generate fluorescent complementary RNA (cRNA) targets for hybridization with CHO-specific oligonucleotide arrays. Labeling and hybridization were performed according to the manufacturer’s instructions. Briefly, 200 ng of total RNA were used for reverse transcription and the subsequent cRNA synthesis and labeling reaction with either cyanine 3 (Cy3)- or cyanine 5 (Cy5)-labeled cytidine triphosphate (CPT). After purification of labeled cRNA using the RNeasy Mini Kit (Qiagen, Venlo, The Netherlands), yield and labeling efficiency was determined using the NanoDrop 1000 sprectrophotometer (Thermo Fisher Scientific, Waltham, MA, USA). The labeling efficiency was >22 pmol Cy3 or Cy5 per μg cRNA for all samples. The cRNA of the appropriate sample and the common reference (pooled RNA from all samples) were mixed and fragmented using the Agilent Gene Expression Hybridization Kit and transferred to the microarray slide. Hybridization was performed at 65 °C for 17 h. After washing, the slides were scanned at 5-μm resolution using an Agilent microarray scanner G2565AB.

The scanned images were processed using the Agilent Feature Extraction 11.0 software. Background correction, normalization, and statistical analysis were performed as previously described (Graf et al. 2008). The resulting *p* values were adjusted for multiple testing using the method of Benjamini and Yekutieli (Reiner et al. 2003).

### Quantitative reverse transcription PCR

MicroRNA and mRNA expressions were measured using the miScript PCR system (Qiagen, Venlo, The Netherlands) which allows the parallel quantification of mature miRNAs and mRNAs. Total RNA extracts were converted into complementary DNA (cDNA) using the miScript II RT Kit (Qiagen) according to the manufacturer’s instructions. Quantitative real-time PCR (qPCR) was performed on a MiniOpticon real-time PCR detection system (Bio-Rad, Hercules, CA, USA) using the miScript SYBR Green PCR Kit (Qiagen) according to the supplier’s manual. To improve the reliability of the assay, the expression of each miRNA was normalized using two internal references (cgr-miR-185-5p and *Actr5*). The miRNA cgr-miR-185-5p was used as an internal control previously (Jadhav et al. 2012), and the mRNA *Actr5* showed very stable expression in the microarray experiment across all CHO cell lines used in this study. Additionally, *Actr5* was described as a suitable internal control gene before (Bahr et al. 2009). For mRNA quantification, *Gapdh* and *Actr5* were used as internal reference genes. A 20 μL qPCR reaction mix contained 10 ng cDNA and the appropriate 10 × miScript Primer Assay (Qiagen). All miScript Primer Assays and other primers used in this study are specified in Table S1 (Supplementary material). The PCR was run at 95 °C for 15 min and 40 cycles of 94 °C for 15 s, 55 °C for 30 s, and 70 °C for 30 s. The specificity of the reactions was verified by analyzing the melting curve immediately after the last amplification cycle. The results were evaluated with the software CFX Manager 3.0 (Bio-Rad). Quantification cycle (*C*_q_) values were determined using the “regression” mode. Three biological replicates (samples from three independent cultivations) were analyzed in technical duplicates (two independent qPCR assays). Relative expression ratios were calculated using the software REST 2009 (Pfaffl et al. 2002). The REST 2009 algorithm uses a statistical randomization test to determine the significance of the expression ratio as well as a complex Taylor series to estimate the standard error (SE).

### Target prediction of differentially expressed miRNAs

For miRNA target prediction, the miRWalk database (Dweep et al. 2011) was used. Besides the miRWalk algorithm, this database includes miRNA-mRNA interactions predicted by DIANA-microT (version 3.0), miRanda (August 2010), miRDB (April 2009), PicTar (March 2007), PITA (August 2008), RNA22 (May 2008), RNAhybrid (version 2.1), and TargetScan (version 5.1). Only interactions predicted by more than half of these programs were considered. The database miRTarBase 4.5 (Hsu et al. 2014) was used to identify potential targets based on validated miRNA-target interactions in human, mouse, or rat. Pearson correlation was analyzed between miRNA expression levels (qRT-PCR data) and mRNA expression levels (microarray data). Student’s *t* tests were used to identify significantly negatively correlated miRNA-mRNA pairs. The resulting *p* values were corrected for multiple testing according to Benjamini and Hochberg (Benjamini and Hochberg 1995).

## Results

### Steady-state cultivation

**Fig. 1:**
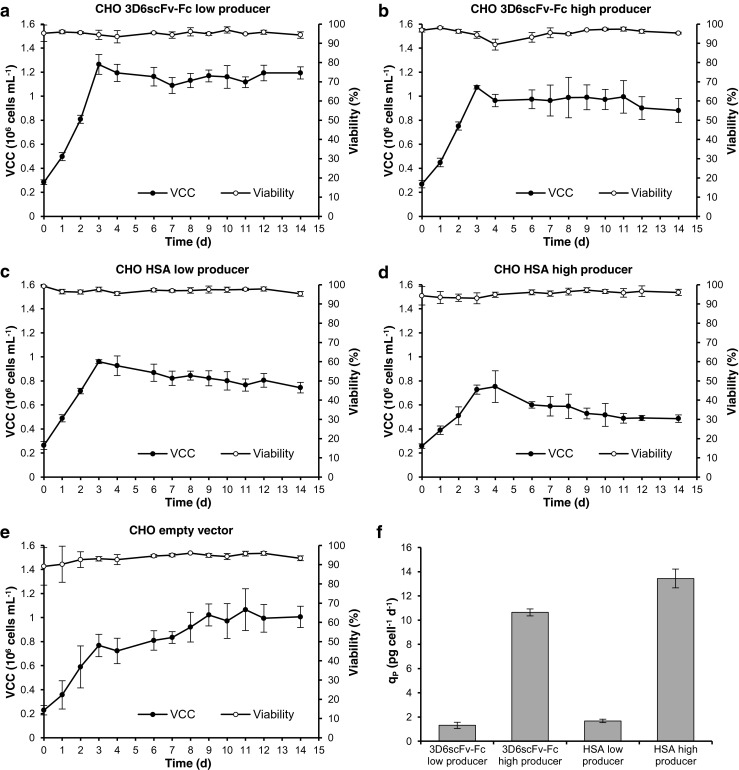
Time courses of steady-state cultivations. Viable cell concentration and viability of **a** CHO 3D6scFv-Fc low producer, **b** CHO 3D6scFv-Fc high producer, **c** CHO HSA low producer, **d** CHO HSA high producer, and **e** CHO empty vector (non-producer). **f** Specific product secretion rate q_P_ in steady-state. Cells were cultivated in a 0.8-L cell culture bioreactor. After 3 days of batch cultivation, the process was switched to continuous cultivation (dilution rate D = 0.5 d^−1^). The culture volume was maintained at a constant level of 400 mL. Data represent mean values of three independent cultivations (*error bars* SD)

The CHO cell lines were cultivated in a continuous process (chemostat) to establish steady-state conditions. After a batch phase of 3 days, the process was switched to continuous operation with a constant dilution rate D of 0.5 d^−1^. The viable cell concentration remained constant during continuous operation (Fig. 1a–e). Consequently, the specific growth rate μ was equal to the dilution rate *D*. Also, the residual glucose, glutamine, lactate, and ammonium reached constant concentrations (Fig. S1a–e, supplementary material), confirming the establishment of steady-state conditions. The specific product secretion rates q_P_ were considerably (eightfold) different between high and low producers but similar for 3D6scFv-Fc and HSA (Fig. 1f). Samples for miRNA and mRNA expression analysis were harvested on day 14 after more than five volume changes.

**Fig. 2:**
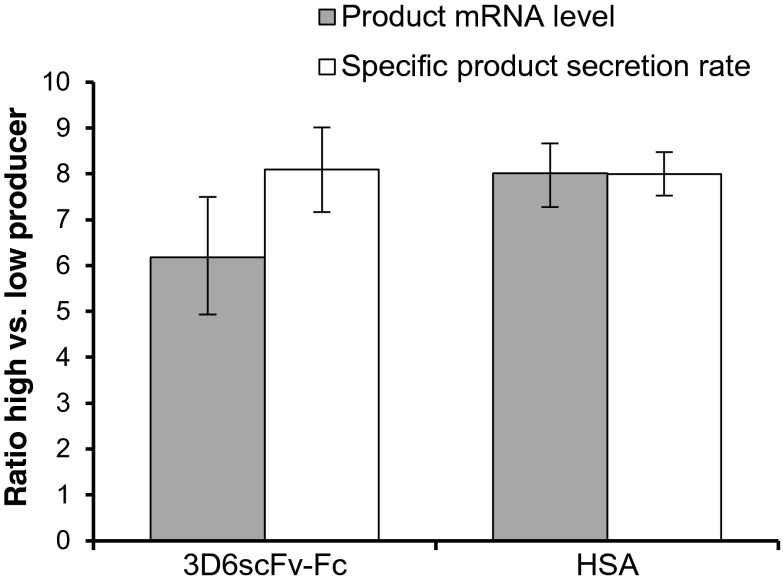
Correlation of product mRNA level and specific product secretion rate q_P_. The diagram shows the ratios of product mRNA and q_P_ of 3D6scFv-Fc and HSA high producers to low producers. Transcript levels were determined by qRT-PCR. Data represents mean values of three independent steady-state cultivations (*error bars* SE)

The high producing cell lines had been established by increasing the transgene copy number of the low producers in order to reduce effects caused by clonal variation (Maccani et al. 2014). Consequently, observed differences in miRNA and mRNA expression profile between high and low producers should predominantly be relatable to cellular processes that are involved in the biosynthesis of the recombinant protein including transcription, mRNA processing and translation as well as protein processing and secretion. The product mRNA levels perfectly corresponded to the determined increase of q_P_ between high and low producers (Fig. 2), indicating that there are no limitations at the stage of translation, protein processing, or secretion. However, differential gene expression between the high and low producers may reflect the adaptations necessary for the cell to handle the higher recombinant protein load.

### MicroRNA expression screening by microarray analysis

**Fig. 3:**
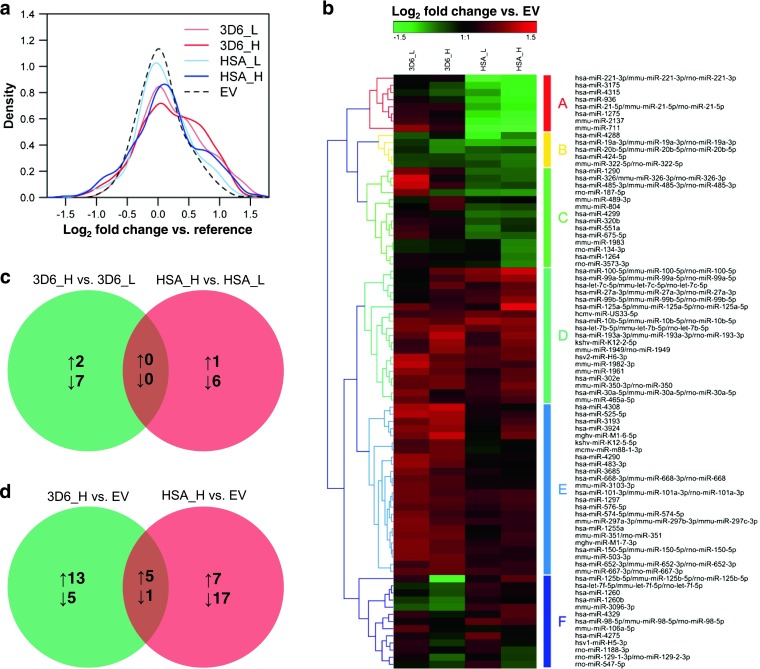
Comparative microRNA profiling using microarray analysis. Total RNA samples of five CHO cells lines from steady-state cultivations (*n* = 3) were analyzed. 3D6_L, CHO 3D6scFv-Fc low producer; 3D6_H, CHO3D6scFv-Fc high producer; HSA_L, CHO HSA low producer; HSA_H, CHO HSA high producer; EV, CHO empty vector (non-producer). **a** Density plot of the log_2_ fold change miRNA expression between each cell line and a common reference pool (mean values, *n* = 3). **b** Hierarchical clustering of 83 significantly differentially expressed mature miRNAs (adj. *p* < 0.05 and fold change > 1.5) based on log_2_ fold changes between producers and non-producer. Commonly and exclusively upregulated or downregulated miRNAs were determined using Venn diagrams. The number of significantly differentially expressed miRNAs of **c** 3D6scFv-Fc high producer versus 3D6scFv-Fc low producer and HSA high producer versus HSA low producer and **d** 3D6scFv-Fc high producer versus non-producer and HSA high producer versus non-producer are illustrated. *upward arrow* upregulated, *downward arrow* downregulated

Expressed and differentially regulated miRNAs were initially identified using a miRNA microarray platform containing probes against 2,367 mature miRNAs from human, mouse, and rat. In total, 320 miRNAs were significantly expressed (signal intensity > background intensity + 2 × standard deviation) in the five CHO cell lines. For these miRNAs, log_2_ fold changes against a common reference pool were calculated and the distribution was analyzed (Fig. 3a). The results showed increased miRNA levels in the producing cell lines compared to the non-producer, where the shift was statistically significant (Student’s *t* test, *p* < 0.05) for 3D6scFv-Fc low producer and 3D6scFv-Fc high producer. Comparing high producers with low producers as well as producers with non-producer, a total of 83 non-redundant miRNAs were significantly differentially expressed (adj. *p* < 0.05 and fold change > 1.5) in at least one of the comparisons. These miRNAs were analyzed using hierarchical clustering (Fig. 3b). The heat map shows that 3D6scFv-Fv expression predominantly led to an upregulation of miRNA expression (cluster D and E). In contrast, a large fraction of miRNAs was downregulated in both HSA producers (clusters A, B, and C). Especially, the miRNAs of cluster A were highly affected by HSA expression. However, comparing the high producers with the low producers, no significantly differentially expressed miRNA (adj. *p* < 0.05 and fold change > 1.5) was commonly upregulated or downregulated for both model proteins (Fig. 3c). Interestingly, more miRNAs were significantly downregulated than upregulated between the individual high and low producers, although we observed a global increase of miRNA levels in the producing cell lines relative to the non-producer. Between high producers and non-producer, five significantly differentially expressed miRNAs were commonly upregulated and one was commonly downregulated (Fig. 3d).

### Differential miRNA expression by qRT-PCR

**Fig. 4:**
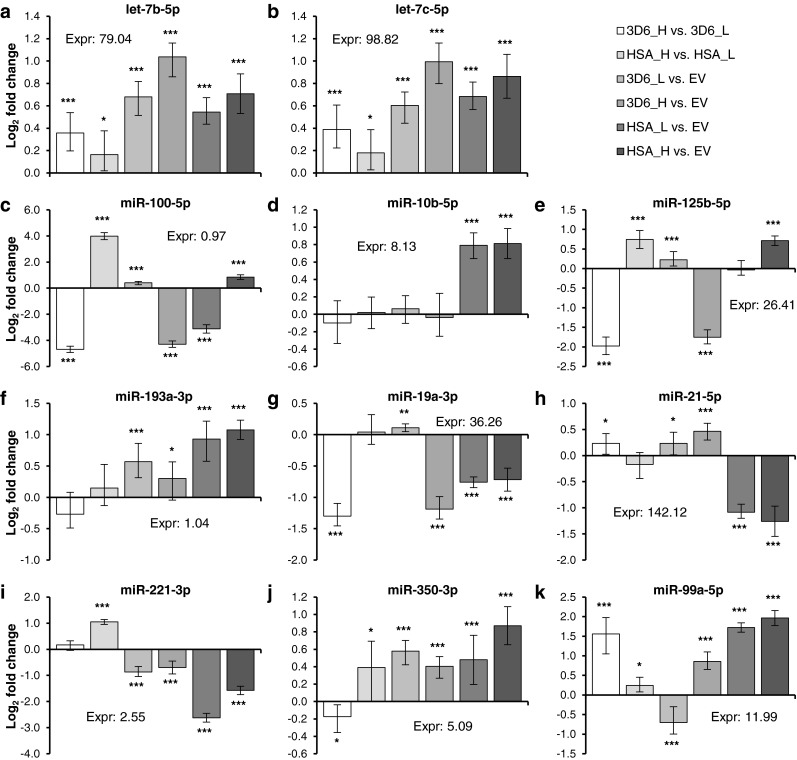
Differentially expressed miRNAs determined by qRT-PCR. 3D6_H, CHO 3D6scFv-Fc high producer; 3D6_L, CHO 3D6scFv-Fc low producer; HSA_H, CHO HSA high producer; HSA_L, CHO HSA low producer; EV, CHO empty vector (non-producer). qRT-PCR data were normalized using two endogenous controls (miR-185-5p and *Actr5*). The software REST 2009 was used to calculate relative expression ratios and for statistical analysis (*p < 0.05, **p < 0.01, ***p < 0.005). Data represent mean values of three independent steady-state cultivations (*error bars* SE)

A total of 14 mature miRNAs from the microarray study were selected for qRT-PCR. The selection was based on the observed most significantly differentially expressed miRNAs between high and low producers as well as between the individual producers and the non-producer including the miRNAs found to be commonly upregulated or downregulated between both high producers and the non-producer. Two of them were human-specific miRNAs (hsa-miR-936 and hsa-miR-3175); one was mouse-specific (mmu-miR-711), and the others showed a 100 % sequence identity between the known mature mouse, human, rat, and Chinese hamster miRNAs according to miRBase 20 (Kozomara and Griffiths-Jones 2014). However, only the 11 conserved miRNAs could be reliably detected by qRT-PCR (Fig. 4). The mature sequences of hsa-miR-936, hsa-miR-3175, and mmu-miR-711 could also not be found in the genome of CHO-K1 (Xu et al. 2011) and the Chinese hamster (Brinkrolf et al. 2013; Lewis et al. 2013) by BLAST search (100 % identity) using the Chinese hamster genome database (Hammond et al. 2012), indicating that those Chinese hamster RNAs leading to a signal on the microarrays are different from the human and mouse miRNAs. In addition, there are no similar Chinese hamster miRNAs which are included in miRBase 20 that could have cross-hybridized to the microarray probes of hsa-miR-936, hsa-miR-3175, and mmu-miR-711.

The miRNAs let-7b-5p and let-7c-5p were upregulated in all producing cell lines compared to the non-producer, and the expression levels correlated with the productivity. Comparing the 3D6scFv-Fc high and low producers, miR-99a-5p was significantly (*p* < 0.005 and fold change > 1.5) upregulated and miR-100-5p, miR-125b-5p, and miR-19a-3p were significantly downregulated. Of these, miR-100-5p and miR-125b-5p showed the inverse effect between HSA high and low producers, where they were significantly upregulated.

### Identification of potential miRNA-mRNA interactions

In order to generate hypotheses about the biological function of the 11 conserved miRNAs analyzed by qRT-PCR, potential target mRNAs were determined based on computational target prediction algorithms and experimentally validated targets, combined with the identification of miRNA-mRNA pairs that show a negatively correlated expression profile. Computational miRNA target prediction was conducted using miRWalk (Dweep et al. 2011), DIANA-microT (Maragkakis et al. 2009), miRanda (John et al. 2004), miRDB (Wang 2008), PicTar (Krek et al. 2005), PITA (Kertesz et al. 2007), RNA22 (Miranda et al. 2006), RNAhybrid (Rehmsmeier et al. 2004), and TargetScan (Lewis et al. 2005). To reduce the false positive rate, only mRNAs predicted by five or more of these nine algorithms were considered. In a second approach, potential targets were obtained from miRTarBase 4.5 using the experimentally validated miRNA-mRNA interactions in human, mouse, or rat.

The mRNA microarray experiment revealed a total of 2,842 genes which were significantly differentially expressed (adj. *p* < 0.05 and fold change > 1.5) in at least one comparison between the five cell lines analyzed in this study. Pearson correlation coefficients (PCC) were computed for the 11 differentially expressed miRNAs (expression levels from qRT-PCR) and the 2,842 differentially expressed mRNAs (expression levels from microarray experiment). Identified potential miRNA-mRNA interactions that showed a negative correlation (PCC < −0.5 and adj. *p* < 0.05) are listed in Table 1. Fisher’s exact test was used to analyze whether negatively correlated targets are significantly (*p* < 0.05) enriched. For the computationally predicted targets, no significant enrichment was observed (Table 2). Regarding the experimentally validated targets, negatively correlated ones were significantly enriched for miR-21-5p and miR-99a-5p (Table 2). Kernel density plots were computed to visualize and compare the distribution of the PCCs for validated targets, predicted targets, and total differentially expressed mRNAs (Fig. S2, supplementary material). Generally, a shift to negative PCCs was observed. However, the shifts were only statistical significant (Student’s *t* test, *p* < 0.05) for the validated targets of miR-10b-5p, miR-21-5p, and miR-125b-5p as well as the predicted targets of let-7c-5p. Hence, these results indicate a considerable degree of false positives within the discovered miRNA-mRNA interactions.

**Table 1:** Identified negatively correlated potential targets of differentially expressed miRNAs

Mature miRNA	Potential target mRNAs^a^
let-7b-5p	*Adam8*, *Ammecr1l*, *Atad3a*, *Cpsf3l*, *Ddx26b*, *Ddx41*, ***Dmd***, *Dnmbp*, *Eef1e1*, *Fam49b*, ***Gltpd1***, *Iars*, ***Igf2bp3***, *Lingo1*, ***Lman2***, ***Mars2***, *Nid1*, *Prss22*, *Ptgs2*, *Slc30a4*, ***Syncrip***, *Taf5*, ***Tmem65***, *Tmprss11f*, *Txndc5*, *Zadh2*, *Zbtb5*
let-7c-5p	*Adam8*, *Ammecr1l*, *Atad3a*, *Bzw1*, *Col4a1*, *Ddx18*, *Ddx26b*, *Fkbp10*, *Gltpd1*, *Golt1b*, *Iars*, *Igf2bp3*, *Jarid2*, *Lingo1*, *Lman2*, *Mars2*, *Pld3*, *Plxna2*, *Prss22*, *Rb1*, *Slc30a4*, *Slk*, *Stk24*, *Syncrip*, *Tmprss11f*, *Txndc5*, *Vps25*, *Zbtb5*
miR-100-5p	–
miR-10b-5p	*Acly*, *Ap3m1*, *Arhgap18*, *Col4a1*, *Ctdspl*, *Eif1*, *Glod4*, *Gpc1*, *Hivep2*, *Idh3a*, *Igf2r*, *Igfbp4*, *Klhdc7a*, *Lrrc16a*, *Lrrc59*, *Mboat1*, *Nkiras2*, *Ormdl1*, *Ppp1r9b*, *Rap2a*, *Rhobtb2*, *Sdc1*, *Slc25a30*, *Stk4*, *Tox4*, *Txndc16*, ***Ube2z***
miR-125b-5p	*6430548M08Rik*, *Ak3*, *Akap1*, *Ankrd13b*, *Arrb1*, *BC003266*, ***Cd320***, *Cln6*, *Cspg4*, *Cyp2c55*, *Cyyr1*, *Dusp3*, *Dynlt3*, *Ebpl*, *Fbn1*, *Fbxw4*, *Fgfr2*, *Gbf1*, *Ghdc*, *Gpc6*, *Gsn*, *Hspd1*, *Icmt*, *Ier3*, *Ilvbl*, ***Jub***, *Ldb1*, ***Lss***, *M6pr*, *Mamdc2*, *Map3k1*, *Mgat5*, *Myt1*, *Ngly1*, *Nup50*, *Osgepl1*, *Pde1a*, *Phex*, *Ppwd1*, *Rere*, *Sept3*, *Slc27a6*, *Snx8*, *St8sia4*, *Stat5b*, *Taf15*, *Thop1*, *Tmem180*, *Tmem201*, *Tnfsf4*, *Tspan9*, *Zmym3*
miR-193a-3p	*Abcc3*, *Acpl2*, *Ap2b1*, *Avpi1*, *Bicd2*, *Ccdc134*, *Cyb561d2*, *Dnajc7*, *Faf2*, *Fzd4*, *Gnat2*, *Igf2bp3*, *Igf2r*, *Ing5*, *Kdelc1*, *Kras*, *Lamc2*, *Lrrc16a*, *Mmp14*, *Nkiras2*, *Npepps*, *Phf21b*, *Slc4a3*, *Slmap*, *Spsb4*, *Tmed3*, *Vps37b*
miR-19a-3p	*Abhd10*, *Ahrr*, *Atp10a*, *Bmp2k*, *Depdc1b*, *Dsel*, *Icmt*, *Mdfic*, *Mid1ip1*, *Pde5a*, *Pdik1l*, *Pls3*, *Rras2*, *Snx7*, *Stx12*, *Timp2*, *Tmem50a*, *Zeb2*
miR-21-5p	*Aftph*, *Alx1*, *Ank2*, *Ankrd28*, *Arhgap24*, ***Atp11b***, *B3galnt1*, *Bmpr1b*, *Boc*, *Ccdc117*, *Cd44*, *Cryab*, *Ctdsp2*, *Dmd*, *Dock4*, *Dse*, *Dync1li2*, *Elf2*, ***Elovl7***, *Entpd5*, *Fbxl17*, *Fubp1*, *Glcci1*, *Gng12*, *Gpam*, *Grsf1*, *Hoxa9*, *Icam1*, ***Mbnl1***, *Mthfd2*, *Nbea*, *Nek1*, ***Nfib***, *Nkiras1*, ***Pdcd4***, *Phf20l1*, *Pias3*, *Pkd2*, *Postn*, *Ppap2a*, *Ptx3*, *Pura*, *Rabgap1l*, *Rbms3*, *Rnf11*, *Rnf167*, *Rpa2*, *Rufy3*, *Sash1*, *Smap2*, ***Srpk2***, ***Taf5***, *Timp2*, ***Trim33***, *Ttc33*, *Uso1*, *Wwc2*, *Zbtb38*, *Zfp110*, *Zfp112*, *Zfp367*
miR-221-3p	*2900011O08Rik*, *Ank2*, *Ankrd28*, *Bmp2k*, *Bmpr1a*, *Capn7*, *Carhsp1*, *Casp9*, *Ccnd2*, *Cd44*, ***Cdkn1b***, *Cxcr7*, *Eaf1*, *Eif4g3*, *Elavl2*, *Eya4*, *Fhl1*, *Figf*, *Glud1*,*Gnptab*, *Gpbp1*, *Icam1*, *Mbnl1*, *Mdfic*, *Nkiras1*, *Nt5dc2*, *Pak1*, ***Pdik1l***, *Phf21a*, *Pkd2*, *Plscr4*, *Ptx3*, *Rfx7*, *Rpl15*, *Sema3b*, *Sema3e*, *Sh3d19*, *Shmt2*, *Slc25a12*, *Slc33a1*, *Slc4a7*, *Sqstm1*, *Ssbp2*, *Stmn1*,*Tapbp*, *Tmem140*, *Tmem176b*, *Wwc2*
miR-350-3p	–
miR-99a-5p	*Ap2b1*, *Apex1*, *Arhgap22*, *Col4a1*, *Ctdspl*, *Ddhd1*, *Ddx18*, *Lman2*, *Nfe2l1*, *Ormdl1*, *Rb1*, *Scpep1*, *Serpine1*, *Smarca5*, *Sucla2*, *Trib1*, *Zfp689*

^a^Computationally predicted miRNA targets, experimentally validated miRNA targets in human, mouse, or rat (underlined), or determined by both methods (bold)

**Table 2:** Enrichment analysis of negatively correlated miRNA targets

miRNA	Number of negatively correlated differentially expressed genes^a^	Number of differentially expressed targets		Number of negatively correlated differentially expressed targets		Odds ratio (OR)^b^		*p* value (Fisher’s exact test)^c^	
		Predicted	Validated	Predicted	Validated	Predicted targets	Validated targets	Predicted targets	Validated targets
let-7b-5p	237	169	205	19	16	1.392	0.931	0.200	0.896
let-7c-5p	314	162	19	24	5	1.400	2.874	0.158	0.052
miR-100-5p	11	17	44	0	0	–	–	–	–
miR-10b-5p	860	57	28	19	12	1.152	1.728	0.663	0.153
miR-125b-5p	514	316	56	50	11	0.851	1.107	0.353	0.727
miR-193a-3p	710	93	0	28	0	1.293	–	0.275	–
miR-19a-3p	243	200	10	18	0	1.058	–	0.794	–
miR-21-5p	825	122	115	41	46	1.237	1.630	0.309	0.016
miR-221-3p	647	159	42	47	14	1.423	1.696	0.053	0.137
miR-350-3p	0	264	0	0	0	–	–	–	–
miR-99a-5p	925	10	24	6	13	3.107	2.448	0.088	0.030

^a^2,842 differentially expressed genes in total

^b^Indicates the degree of enrichment/depletion. OR > 1, negatively correlated differentially expressed targets are overrepresented. OR < 1, negatively correlated differentially expressed targets are underrepresented

^c^Significance of enrichment/depletion

## Discussion

### Comparable conditions by steady-state cultivation

A prerequisite for a comparative physiological analysis of different cell lines is the generation of samples under comparable and defined conditions in order to obtain reproducible and meaningful information. In simple batch cultures, the physicochemical conditions are very dynamic and have a considerable impact on the cell’s transcriptome (Hernandez Bort et al. 2012; Koh et al. 2009). Koh et al. observed significant changes in miRNA expression even within the exponential growth phase of batch cultivated HEK-293 cells. This clearly shows that in batch cultivations, the time of sampling is crucial for the outcome of omics studies that compare different cell lines. Consequently, the chemostat with its defined and constant environment is the ideal setup for such experiments (Hoskisson and Hobbs 2005). Chemostat cultures have been used to grow CHO cells under steady-state condition for more than two decades (Hayter et al. 1992). However, as the standard industrial bioprocess is fed-batch, steady-state cultivation was not applied in recent CHO omics studies.

### Product dependency of miRNA expression profile

The most striking observation in our study was the divergence in differentially regulated miRNAs between the cell lines producing different proteins. We observed a general increase of miRNA expression levels in the producing cell lines, which indicates that miRNAs play an important role in the regulation of processes involved or caused by recombinant protein synthesis and secretion. Several miRNAs were differentially expressed comparing high, low, and non-producing CHO cell lines. However, significant differences in miRNA expression were predominantly seen between producers and non-producers as well as between 3D6scFv-Fc and HSA producers, rather than between high and low producers. Furthermore, no significantly differentially expressed miRNA was commonly upregulated or downregulated comparing high and low producers for both model proteins. These results suggest that the reaction of CHO cells to recombinant protein expression strongly depends on the particular product, which would also explain the low level of consensus observed between previously published studies investigating the transcriptome of different CHO production cell lines in relation to high productivity (Vishwanathan et al. 2014). Different proteins require specific cellular aid with folding, glycosylation, and bonding depending on their structure. The exerted stress may well initiate differential responses and consequently affect the regulation of both miRNAs and mRNAs that provide the cells with the capacity to handle the various types of stress. As the mRNA expression of the product genes correlated well to the cell-specific productivity, it appears that the cellular protein production machinery was able to adapt to these individual requirements without running into limitation. Nevertheless, the contribution of effects caused by clonal variation is unknown and cannot be neglected.

### Impact of confirmed miRNAs

For those differentially regulated miRNAs that were confirmed by qRT-PCR, some consensus could be found in similar previously published studies. In an earlier work, we compared the miRNA expression of an Epo-Fc and an antibody producing cell line to the respective parental CHO cell line (Hackl et al. 2011). We observed an upregulation of miR-10b-5p and downregulation of miR-21-5p in recombinant cell lines, which is in compliance with our results of the HSA producers but not for the 3D6scFv-Fv producers. Additionally, we could already show that miR-21-5p overexpression reduces specific productivity (Jadhav et al. 2012). Consequently, this suggests that a knockdown of miR-21-5p could increase specific productivity. As one of the best studied miRNAs, miR-21-5p has been linked to cell proliferation, apoptosis, and migration (Krichevsky and Gabriely 2009) and even cellular longevity (Dellago et al. 2013). Like miR-21-5p, miR-10b-5p was described as an oncogenic miRNA with a pro-proliferative and anti-apoptotic function (Lin et al. 2012).

In another study, Lin et al. (2011) profiled miRNA expression in four recombinant CHO cell lines expressing the same human IgG and compared them with the parental DG44 cell line. In compliance with our results, they found miR-221-3p being significantly downregulated in the recombinant cell lines. They also observed a downregulation of miR-125b-5p in two clones, as observed in the 3D6scFv-Fc high producer in our study. However, they detected opposed effects for miR-19a-3p and let-7b-5p. In human hepatocellular carcinoma cells, miR-221-3p was found to control cyclin-dependent kinase inhibitor 1B (p27^Kip1^) and cyclin-dependent kinase inhibitor 1C (p57^Kip2^) expression (Fornari et al. 2008). Induction of G1-specific growth arrest by conditional overexpression of p27^Kip1^ resulted in increased specific productivity in CHO (Meents et al. 2002); however, transient overexpression of miR-221 had no significant effect on growth or productivity (Jadhav et al. 2012). This might indicate that miR-221 expression changes in response to cellular requirements, but forced manipulation of its expression does not by itself change the phenotype. miR-125b-5p can act as tumor suppressor or as oncogene, and it was shown to promote apoptosis by suppressing the expression of Bcl-2 family members (Gong et al. 2013).

All miRNAs described above, have been associated with cell growth and/or apoptosis, so that their relation to productivity may only be indirect and therefore difficult to interpret. Hence, further evaluation of the identified target miRNAs is required.

### The challenge of high-throughput miRNA target identification

Identifying the biological function of a miRNA is still a major challenge. Due to the complexity and diversity of miRNA-mRNA target interactions, functional screenings using biological methods are very labor-intensive and time-consuming. Hence, reliable computational tools for the prediction of interactions between miRNAs and target mRNAs would be a huge benefit. But the prediction of targets is very challenging as miRNAs recognize specific sequences with only partial complementarity. However, although perfect pairing between nucleotides 2–7 (seed region) and the target site is the most common motif in animals (Pasquinelli 2012), also imperfect seed pairing compensated by extensive pairing of the 3′ end (Vella et al. 2004) and centered pairing (Shin et al. 2010) have been described. Numerous algorithms based on seed pairing, evolutionary conserved sites, secondary structure of the 3′ UTR, and thermodynamic calculations have been developed for computational target prediction. Many of these algorithms consider cross-species conservation to reduce false positive rates (Maziere and Enright 2007). However, by applying a novel biochemical approach known as high-throughput sequencing of RNA isolated by cross-linking immunoprecipitation (HITS-CLIP), it was shown that a considerable fraction (40 %) of all functional target sites is not conserved (Ellwanger et al. 2011). The lack of agreement between the results of different computational methods as well as the high false positive and false negative rates clearly show the complexity of target prediction in mammalian cells (Liu et al. 2014; Ritchie et al. 2009). In CHO research, the reliability of the currently available target prediction tools is additionally impaired as the CHO or Chinese hamster genome has not been included in any of them yet. Hence, the results rely on an assumed high degree of conservation of miRNA and target mRNA interactions between Chinese hamster and mouse, rat, or human.

In mammalian cells, various studies suggest that miRNAs predominantly act by decreasing target mRNA levels (Baek et al. 2008; Guo et al. 2010; Hendrickson et al. 2009). This supports the frequently used approach of correlating miRNA and mRNA expression levels to identify negatively correlated pairs, which we also applied here. We analyzed whether negatively correlated miRNA-mRNA pairs are enriched within computationally predicted and experimentally validated targets. However, a statistically significant enrichment could only be observed within the validated targets for two of the 11 analyzed miRNAs. This approach also assumes that the target mRNA is predominantly regulated by the miRNA and not at a different stage of gene expression. In addition, it has been shown that most miRNAs are only discernibly active above a certain expression level (Mullokandov et al. 2012). Furthermore, there are several factors that can affect miRNA-binding efficiency to a specific target mRNA, including competition with RNA-binding proteins (Kedde et al. 2007), miRISC cofactors (Neumüller et al. 2008) or modification of argonaute proteins (Johnston and Hutvagner 2011). In addition, mRNAs can contain multiple target sites for a single miRNA and also target sites for several miRNAs which suggests even more complex regulatory mechanisms by miRNAs (Liu et al. 2014). Together, this indicates that many targets most likely remain undiscovered by the simple assumption of an inverse relationship between the expression levels of a miRNA and its target mRNAs.

In conclusion, this is the first report of miRNA expression data of recombinant CHO cell lines cultivated under steady-state conditions. Cell lines that express heterologous proteins appear to have higher levels of mature miRNAs in general, which suggests that miRNAs play a crucial role in recombinant CHO cell lines. However, comparing the miRNA expression profiles of different CHO cell lines, both from this study and published results, revealed little consensus. This indicates that the reaction of CHO cells to the overexpression of heterologous proteins is strongly protein and/or clone dependent. Hence, cell engineering approaches to improve recombinant protein production may also be product- and/or clone-specific and not generally applicable.

Identifying miRNAs and their target mRNAs is crucial for a better understanding of biological processes in CHO cells, and there is no doubt that high-throughput miRNA and mRNA profiling can deliver valuable information. Additional results from proteome analyses would also be beneficial to obtain a comprehensive picture (Baek et al. 2008). As the regulatory mechanisms of miRNAs are very complex and today’s computational target prediction tools are inefficient, it is still a major challenge to retrieve correct and meaningful results from high-throughput omics data. Hence, reliable computational miRNA target prediction tools that also include the CHO genome are urgently needed. This also requires thoroughly defined 3′ UTR boundaries in the CHO genome as this highly influences the outcome of miRNA target prediction (Ritchie et al. 2009). In addition, experimentally validated miRNA targets in CHO cells need to be collected in order to finally facilitate functional analysis of high-throughput miRNA expression data.

## Electronic supplementary material

Below is the link to the electronic supplementary material.

ESM 1 (PDF 768 kb)
