# Supplementary material for: Identification of microRNAs specific for high producer CHO cell lines using steady-state cultivation
Source: Appl Microbiol Biotechnol. 2014 Jul 23;98(17):7535–48. doi: 10.1007/s00253-014-5911-4 (PMC4139590; doi:10.1007/s00253-014-5911-4)
Supplement: Supplementary file 1 — (PDF 768 kb) [file 253_2014_5911_MOESM1_ESM.pdf]

Identification of microRNAs specific for high producer CHO cell lines using steady-state cultivation

Andreas Maccani[1,2], Matthias Hackl[2], Christian Leitner[3], Willibald Steinfellner[2], Alexandra B. Graf[1,4], Nadine E. Tatto[1], Michael Karbiener[5], Marcel Scheideler[5], Johannes Grillari[2], Diethard Mattanovich[1,2], Renate Kunert[1,2], Nicole Borth[1,2], Reingard Grabherr[2], Wolfgang Ernst[1,2]

[1] Austrian Centre of Industrial Biotechnology (ACIB GmbH), Muthgasse 11, 1190 Vienna, Austria

[2] Department of Biotechnology, VIBT – BOKU – University of Natural Resources and Life Sciences Vienna, Muthgasse 18, 1190 Vienna, Austria

[3] Department of Food Science and Technology, VIBT – BOKU – University of Natural Resources and Life Sciences Vienna, Muthgasse 18, 1190 Vienna, Austria

[4] School of Bioengineering, University of Applied Sciences FH-Campus Vienna, Muthgasse 56, 1190 Vienna, Austria

[5] Institute for Genomics and Bioinformatics, Graz University of Technology, Petersgasse 14, 8010 Graz, Austria

Correspondence:

Andreas Maccani, Tel: +43 1 47654 6908, Email: andreas.maccani@acib.at

Wolfgang Ernst, Tel: +43 1 47654 6944, Email: wolfgang.ernst@boku.ac.at

Table S1: Primers used for qRT-PCR

Target	Primer sequence (5’–3’) or Qiagen miScript Primer Assay
Actr5 sense	CTCCTTCCAGGTTCAGCTTG
Actr5 antisense	GGCACAATGTTCCTTGAGGT
Gapdh sense	GTAAGAAGCCCACCCTGGA
Gapdh antisense	GTGAGGGAGATGATCGGTGT
3D6scFv-Fc sense	CCCAAGCTGCTGATCTACAA
3D6scFv-Fc antisense	GATGGTCAGGGTGAACTCG
HSA sense	CCTGGAAGTGGACGAGACATAC
HSA antisense	GTCTGCTTCTTGATCTGCCTTT
let-7b-5p	MS00001225
let-7c-5p	MS00005852
miR-100-5p	MS00032214
miR-10b-5p	MS00032249
miR-125b-5p	MS00005992
miR-19a-3p	MS00001302
miR-185-5p	MS00001736
miR-193a-3p	MS00001785
miR-21-5p	MS00011487
miR-221-3p	MS00032585
miR-350-3p	MS00007938
miR-99a-5p	MS00033117

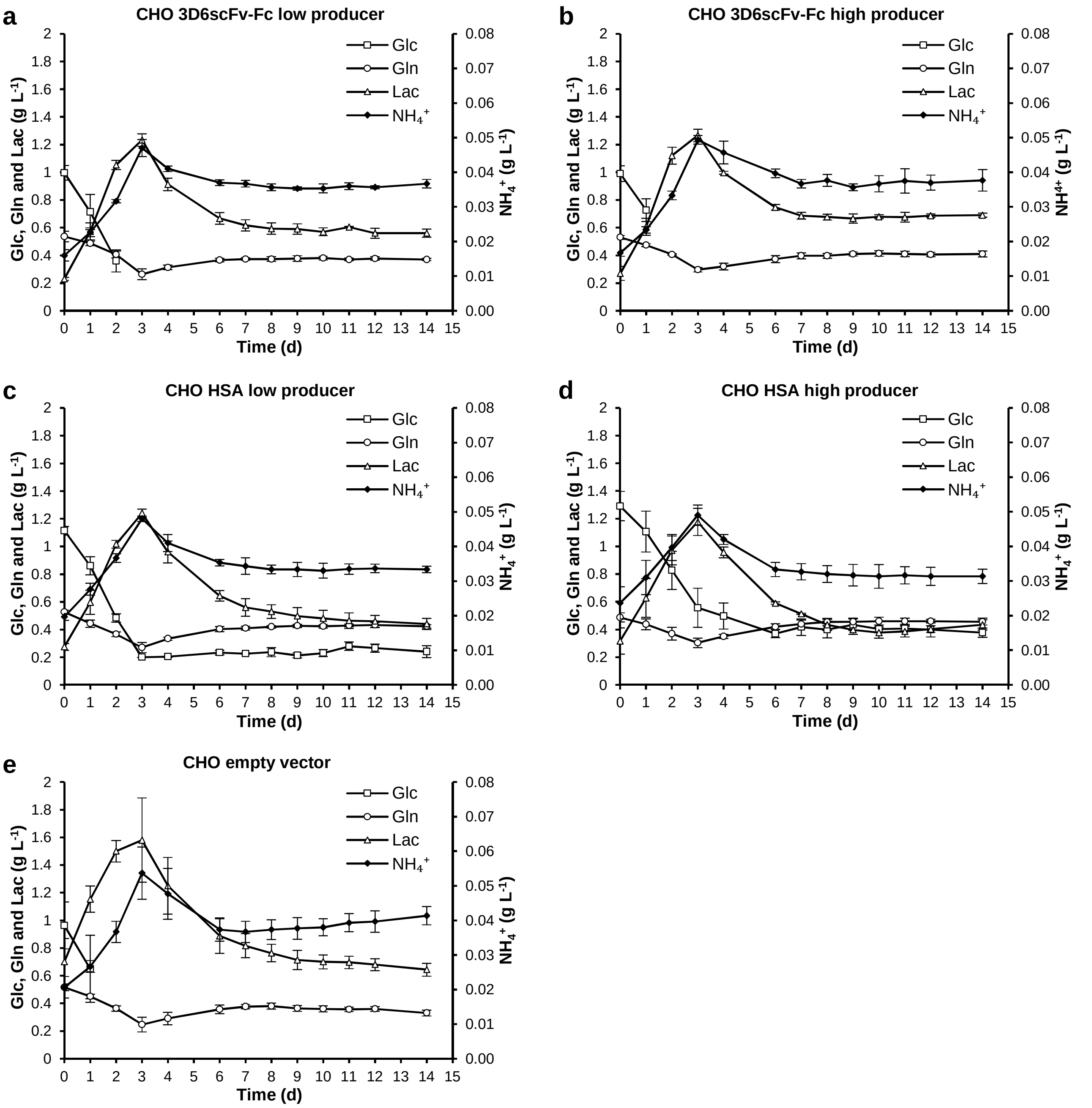

Fig. S1 Time courses of steady-state cultivations. Glucose (Glc), glutamine (Gln), lactate (Lac) and ammonium (NH_4^+) concentration of (**a**) CHO 3D6scFv-Fc low-producer, (**b**) CHO 3D6scFv-Fc high-producer, (**c**) CHO HSA low-producer, (**d**) CHO HSA high-producer and (**e**) CHO empty vector (non-producer). Cells were cultivated in a 0.8 L cell culture bioreactor. After three days of batch cultivation, the process was switched to continuous cultivation (dilution rate D = 0.5 d^{-1}). The culture volume was maintained at a constant level of 400 mL. Data represent mean values of three independent cultivations (error bars: SD). No data points are shown for glucose concentration below the detection limit of 0.2 g L^{-1}

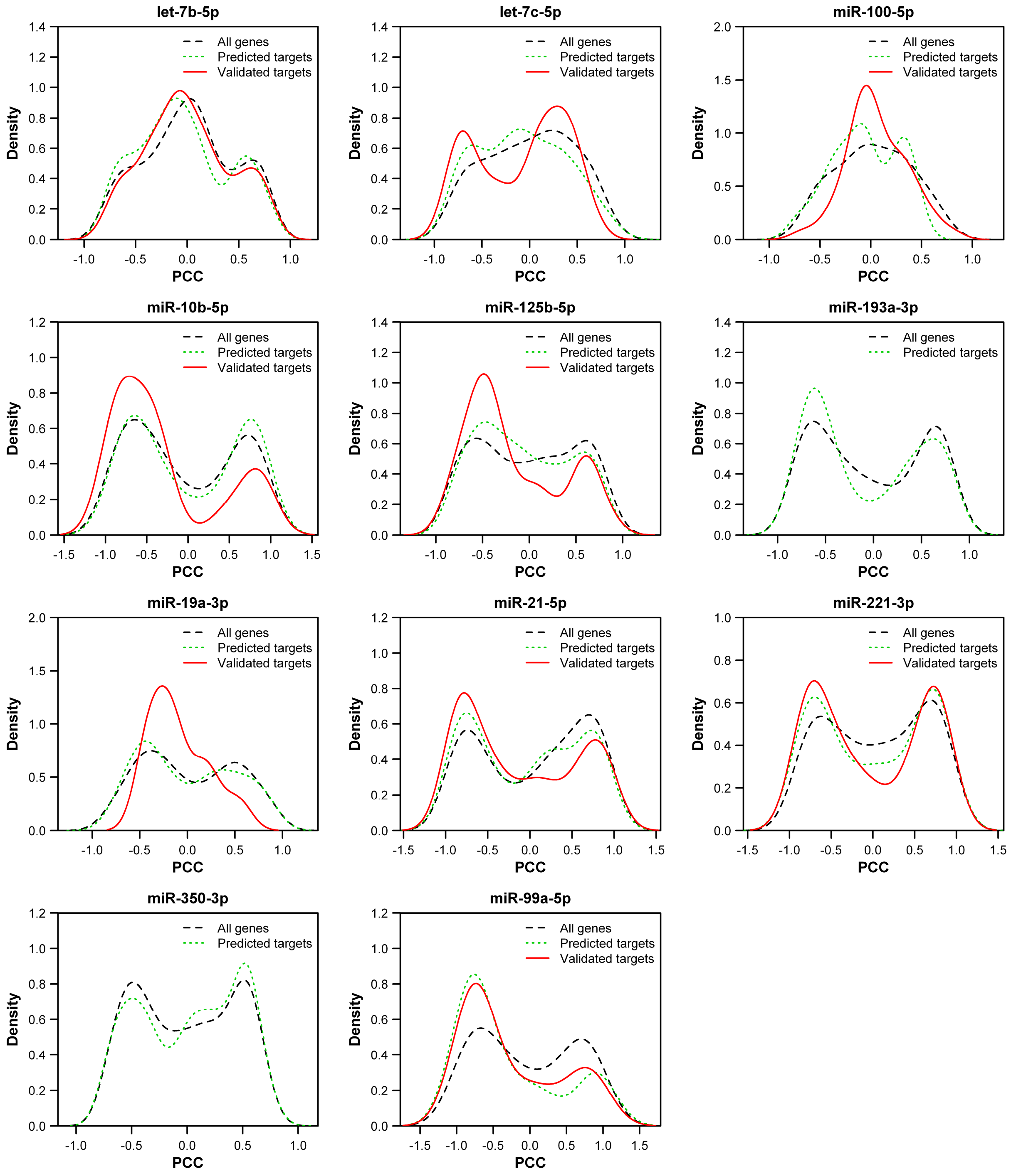

Fig. S2 Distribution of Pearson correlation coefficients (PCC) between miRNAs and validated or predicted targets. Superimposed kernel density plots were computed with equal bandwidths. All genes: 2843 mRNAs which were identified as differentially expressed (adj. $p < 0.05$ and fold change > 1.5) between the cell lines used in this study. Predicted targets: Differentially expressed target mRNAs that were computationally predicted by more than half of nine applied algorithms. Validated targets: Differentially expressed target mRNAs which have been experimentally validated in human, mouse or rat (miRTarBase 4.5)
